# Tremor, finger and hand dexterity and force steadiness, do not change after mental fatigue in healthy humans

**DOI:** 10.1371/journal.pone.0272033

**Published:** 2022-08-10

**Authors:** Francesco Budini, Luciana Labanca, Michael Scholz, Andrea Macaluso

**Affiliations:** 1 Institute of Human Movement Science, Sport and Health, University of Graz, Graz, Austria; 2 Department of Movement, Human and Health Sciences, University of Rome Foro Italico, Rome, Italy; 3 Department of Economics, University of Graz, Graz, Austria; 4 Department of Economics, QED, University of Klagenfurt, Klagenfurt, Austria; Federation University Australia, AUSTRALIA

## Abstract

The effects of mental fatigue have been studied in relation to specific percentages of maximal aerobic or anaerobic efforts, maximal voluntary contractions or the performance of sport specific skills. However, its effects on tremor, dexterity and force steadiness have been only marginally explored. The present work aimed at filling this gap. In twenty-nine young individuals, measurement of postural, kinetic and isometric tremor, pinch force steadiness and finger and hand dexterity were performed before and after either 100 min of mental fatigue or control tasks. During the interventions blood pressure, oxygen saturation and heart rate and perceived effort in continuing the task were recorded every 10 minutes. Tremor was analysed in both time (standard deviation) and frequency domain (position, amplitude and area of the dominant peak) of the acceleration signal. Finger dexterity was assessed by Purdue pegboard test and hand dexterity in terms of contact time in a buzz wire exercise. Force steadiness was quantified as coefficient of variation of the force signal. Postural, kinetic and isometric tremors, force steadiness and dexterity were not affected. Higher oxygen saturation values and higher variability of heart rate and blood pressure were found in the intervention group during the mental fatigue protocol (p < .001). The results provide no evidence that mental fatigue affects the neuromuscular parameters that influence postural, kinetic or isometric tremor, force steadiness and dexterity when measured in single-task conditions. Increased variability in heart rate may suggest that the volunteers in the intervention group altered their alert/stress state. Therefore, it is possible that the alterations that are commonly observed during mental fatigue, and that could have affected tremor, steadiness and dexterity only last for the duration of the cognitive task and are not detectable anymore soon after the mental task is terminated.

## Introduction

Mental fatigue is a psychobiological state caused by prolonged cognitive efforts, characterised by altered electroencephalographic activity and subjective feelings of fatigue [1–3]. While it has been commonly observed that mental fatigue induces a decline in cognitive performance, assessed as an increased reaction time to stimuli and as an increased number of errors in simple mental tasks [4–6], reductions in attention [7, 8] and reductions in task vigilance [9], its effects on exercise performances are in relation to the type of physical task. According to recent literature reviews [10–12], endurance, sport motor skills and decision-making performances seem to be affected, whilst maximal strength, power, and anaerobic work are usually not.

Physical tasks, however, are not limited to activities that require to sustain aerobic or anaerobic efforts, maximal voluntary contractions or the performance of sport-specific skills, therefore, the effects of mental fatigue have also been studied in relation to driving [2, 13], balance and fall prevention [14–19].

It is similarly meaningful to investigate the possible effects of mental fatigue on different everyday activities that require manual dexterity and submaximal force output steadiness, with these being particularly relevant for several professions in which it is required to maintain prolonged cognitive efforts as well as high levels of manual dexterity and precision, e.g. during surgical procedures [20, 21]. The effect of mental fatigue on muscle steadiness has been explored in several recent studies [22–28] by testing force fluctuations during isometric efforts performed at a specific percentage of the maximal voluntary contraction during or after a cognitive effort. Isometric contractions though, have a limited ecological relevance, being neither functional nor dexterity tasks nor fine controlled movements. Indeed, to test these parameters, specific tools (as for example the Purdue board) as well as specific goal-directed exercises resembling normal and professional daily actions have been developed [29–31]. In only a few studies, however, the effects of mental fatigue have been evaluated by adopting testing procedures appropriate to assess goal-directed movements [32] and dexterity [33, 34].

Of these three works, the study by Duncan and colleagues (2015) remains predominantly oriented towards sports practice, as the authors intended to study the effect of mental fatigue on a combination of intermittent anaerobic exercises and motor skills, as common features of some sports. Accordingly, their hand dexterity test consisted in the Minnesota Manual Dexterity Turning Test, which might represent an appropriate assessment for sports requiring good and fast hand-eye coordination, but not for professions demanding a high degree of accuracy. Moreover, the sample size was small (n. 8) and the duration of the mental fatigue protocol adopted was too short (40, minutes) to be comparable with professional requirements that can last several hours [35]. Similarly, the study by Valenza et al. (2020) was conducted on a small sample size (n. 7) and the mental fatigue protocol was of short duration (35 minutes). Rozand et al. (2015) reported that fatigued volunteers performed slower in a speed-accuracy pointing task, independently on the difficulty of the task. The speed-accuracy task test, as the name suggests, requires the participants to perform as fast and as precise as possible a given goal-directed movement, finding the best compromise to optimise the performance. Therefore, also this test does not resemble everyday activities or professional requirements since these activities would not need to be executed as fast as possible, but precisely and with high degree of hand dexterity. One element that can compromise precision and hand dexterity is muscle tremor. To our knowledge, the effects of mental fatigue on tremor was only tested by Budini and colleagues (2014a) who reported that mental fatigue decreases mechanically amplified muscle tremor during sustained knee extension anisometric submaximal contractions. However, the result can represent neither a parameter of accuracy nor a normal physiological condition nor a functional task.

Therefore, overall, the effects of mental fatigue on tremor, force steadiness and functional tasks involving the upper limb during fine controlled movements remain largely unknown.

The aim of the present work is hence to study the effect of mental fatigue on manual and finger dexterity, force steadiness and muscle tremor during upper limb postural and dynamic goal-directed movements and low-intensity isometric pinch contractions.

## Methods

### Participants

Twenty-nine recreationally active individuals: 19 males (25.1±5 years, 72.0±5.2 kg, 1.78±0.06 m) and 10 females (26.6±3 years, 62.3±5.9 kg, 1.75±0.07 m) with no history of neurological or cardiovascular disorders volunteered for the experiment. Volunteers were required to abstain from any strenuous physical activity on the testing day as well as to refrain from taking caffeine-containing substances and smoking within 2 h afore the testing session. Participants were randomly assigned by drawing lot to either an intervention group (n. 15: 9 males, 6 females, 26.7±5 years, 69.0±6.4 kg, 1.76±0.08 m) or a non-intervention control group (n. 14: 10 males, 4 females, 24.4±3 years, 68.3±8.0 kg, 1.74±0.07 m). The study was approved by the Review Board of the University of Rome Foro Italico and written informed consent was obtained from all volunteers before the onset of the experimental procedures. The volunteers were not informed about the real project objective and were told the study investigated the correlation between the ability of maintaining concentration and the performance of different manual tasks. At the end of the testing session, participants were debriefed about the purpose of the study and were asked not to reveal this information to other volunteers.

### Study design and procedures

The participants were requested to attend the laboratory for one single experimental session, lasting about 2 hours and 45 minutes (~25 minutes preparation/familiarisation, ~20 minutes pre-intervention test, 100 minutes intervention, ~20 minutes post-intervention test); during this time the room temperature was monitored and kept at 23.0 ±1.5 degrees C˚ throughout the experiment.

**Fig 1:**
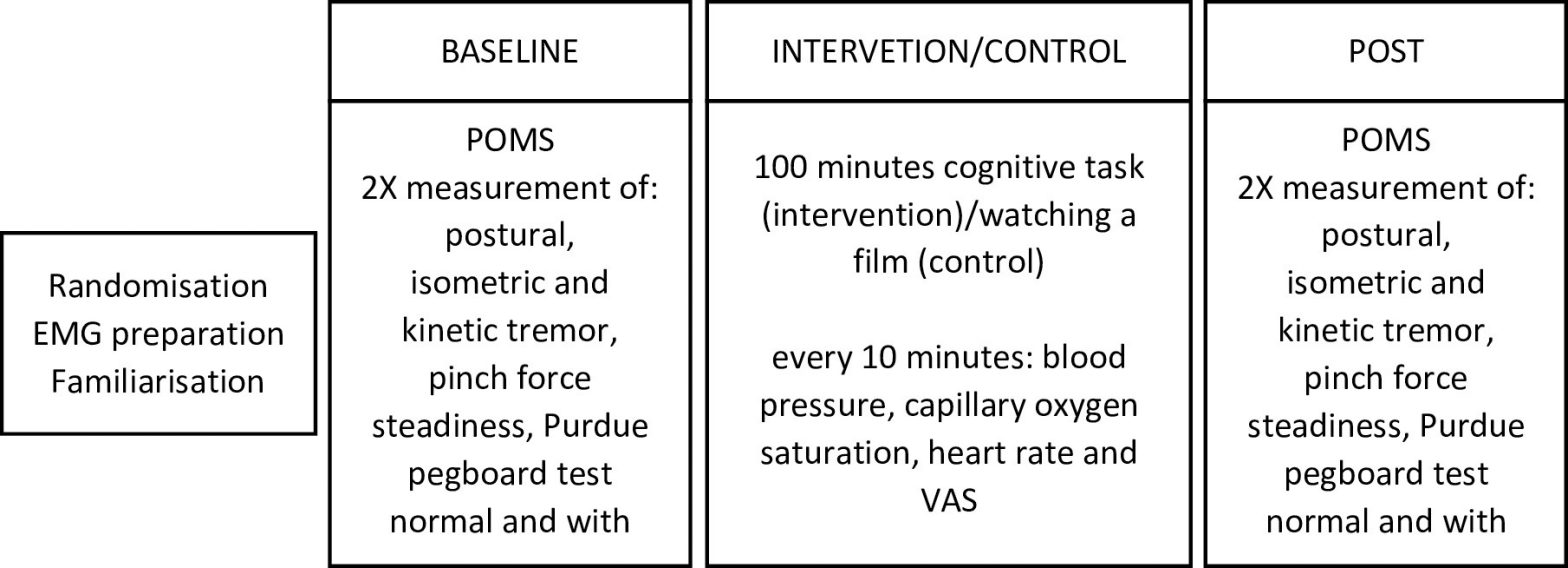
Protocol timeline. POMS = Profile of Mood States; VAS = Visual Analogue Scale.

Protocol timeline is illustrated in Fig 1. Before starting data collection, the volunteers were prepared for EMG recording and completed five familiarization trials for each of the two Purdue tasks and five for the kinetic tremor task (details in the following sections). The experiment consisted in the measurement of postural, isometric and kinetic tremor, pinch force steadiness, Purdue pegboard test and a modified version of it to be performed with tweezers. Each assessment was performed in random order twice before and twice after either 100 minutes of continuative cognitive task (intervention protocol) or 100 minutes during which the volunteers were watching a film (control protocol). For the order of the testing procedures 4 different sequences of trials were used and one of these randomly allocated to each of the volunteers. The sequence itself was previously randomly generated by the experimenters (by drawing lot) and provided the order of procedures for both the baseline and post intervention (the sequence of the procedures for the baseline was always different from the post intervention sequence). Before and every ten minutes during the period the participants seated in front of the computer for either protocol, blood pressure (measured by an electronic blood pressure cuff positioned on the bare arm, approximately 3 cm above the elbow crease) [36], capillary oxygen saturation and heart rate (measured by finger pulse oximetry from the left index finger) were noted. For these measurements the volunteers remained seated with the forearm resting on the table and continued their assigned tasks. Moreover, a visual analogue scale (VAS) printed on a sheet was shown to the participants which were asked to quantify in a 0 to 10 scale, with 0 representing “no fatigue” and 10 representing “extreme fatigue”, their perceived effort exerted in performing the task they were assigned to (cognitive or control). Finally, immediately before and after either protocol, the subjects completed a 32-items Profile Of Mood States questionnaire (POMS) [37] with eight subscales: tension, depression, anger, fatigue, vigor, confusion, happiness and calmness (we used Italian terms [38]). Halfway through the cognitive or control protocol, the volunteers were given 200 ml of water and a snack (25 gr, 100 Kcal, Proteins 1,8 gr, CHO 20.7 gr, Fats 0.9 gr.) that were consumed during the performance of the assigned task, to avoid the onset of thirst or hunger during the subsequent part of the experiment.

### Postural tremor assessment

The volunteers seated on a chair with their forearm supported on the armrest and the wrist joint aligned to its edge, so that the hand was not supported by the armrest; the volunteer was instructed to maintain the hand horizontal (palm down) in line with the supported forearm and the fingers loosely extended and to gaze upon a fix point at 2-m distance. Tremor was recorded for 20 seconds using a 3-axis accelerometer (MPU– 6050, SparkFun Electronics ®) fixed to the dorsal aspect of the hand with the y axis aligned with the third metacarpal bone. Postural recording of tremor was also performed with the arm outstretched at shoulder level.

### Kinetic tremor and hand dexterity assessment

**Fig 2:**
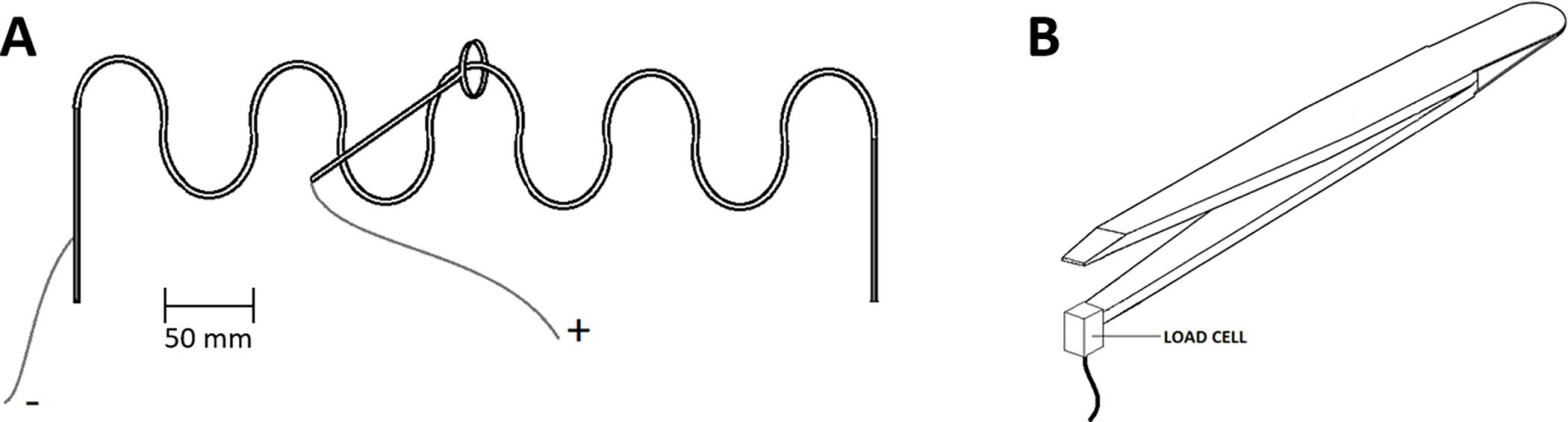
Testing equipment. **A**: The circuit for the kinetic tremor assessment; **B**: Load cell connected to the tweezers for the assessment of pinch force steadiness.

Kinetic tremor was recorded with the same accelerometer (MPU– 6050, SparkFun Electronics ®) fixed to the dorsal aspect of the hand during the performance of a buzz wire circuit: the participant standing in front of a 0.5 m long wire comprising 5 bends of the same size and shape (half-circle ~5 cm diameter) while holding a 20 g wand with a 2 cm diameter metal loop at its extremity (Fig 2A). The volunteers were instructed to follow the bent wire shape with the wand loop engaged in the circuit and complete the circuit from left to right and return trying not to touch it while performing movements of prono-supination only. The contact time between the wand and the circuit was used as index of hand dexterity [39]. For this task, the volunteers had five familiarisation trials before the beginning of the testing session. During the familiarisation trials, the volunteers were invited to find a comfortable posture and a suitable distance from the circuit that would have allowed the performance of the task without moving the feet. Familiarisation sessions were timed and the volunteers were asked to try to complete each trial in about the same time. During the test no restrictions about the execution speed were given although the volunteers were invited to complete the task in approximately the same time they completed it during the familiarisations. The time required for completing the circuit was measured, however, no feedback about execution time was provided and the task was always self-paced.

### Purdue Pegboard test (finger dexterity)

A 30 second, single hand, Purdue Pegboard test was performed with the dominant hand following standard guidelines for this assessment (take one pin at the time from the built-in compartment of the board and place it into the first pin hole located in the pegboard, proceed sequentially and as quick as possible aiming to insert a maximum number of pins). The same test was also repeated in a modified version where the pins needed to be inserted in the holes by using tweezers. As for all the hand dexterity measurements, also for these tasks the volunteers had five familiarisation trials before the beginning of the testing session.

### Pinch force steadiness

Participants were seated on a rigid custom-made chair holding a modified tweezer between their thumb and index fingers at marked points on the tweezer´s arms. A piezoelectric force transducer (Kistler 9203, Winterthur, Switzerland) replaced one of the two extremities of the tweezer (Fig 2B). Pinching force output was assessed isometrically at two different absolute load values (3 and 5 N) sustained for 25 seconds. The force signal was amplified (1K) (Kistler Charge Amplifier Type 5011, Winterthur, Switzerland) and displayed on an oscilloscope (Tectronix TDS 220, Beaverton, USA) positioned in front of the participants where a horizontal cursor provided the visual feedback of the target force value.

### Electromyography

After appropriate skin preparation, surface electromyography was recorded from the first dorsal interosseous (FDI) by concentric detection electrode (CoDe 1.0 B, OT Bioelettronica). A ground bracelet was placed distally on the forearm at the wrist level.

### Cognitive protocol for mental fatigue

The protocol for mental fatigue used in this experiment was based on a switch task paradigm that has already been described in details elsewhere [4]. Briefly, the participant sat in front of a computer where a black cross divided the white screen in four squares. The first stimulus appeared in the top left square and disappeared after either 2500 ms had elapsed or the user responded. After random intervals (150, 600 or 1500 ms) a new stimulus appeared in the top right square and so on clockwise continuously for 100 minutes. Stimuli were letters that could be red or blue and either consonants or vowels. When the stimulus appeared in any of the top squares, the participant was instructed to respond with a right choice (pressing the enter key on the computer keyboard) if it was red and with a left choice (pressing the spacebar on the computer keyboard) if it was blue. When the stimulus appeared in any of the bottom squares, the participant was instructed to respond with a right choice if it was a vowel and with a left choice if it was a consonant.

### Data analysis

The acceleration, force and electromyography were synchronized digitized with a sampling frequency of 2048 Hz, stored on a PC and analysed using custom algorithms developed in Matlab (7.8.0.347 R2009a).

Postural tremor was quantified by examining the standard deviation of the low passed (30 Hz) filtered acceleration signal averaged for the 3 axes and calculated over the last 15 seconds of each postural task. The position and amplitude of the dominant peak within the alpha band in the power spectra of the accelerometer signals, and the area within 0.5 Hz of the peak, were calculated (2048-point, hamming window fast Fourier transform). The kinetic data were similarly analysed after additional high pass filtering at 2 Hz to eliminate the big fluctuations related to voluntary prono-supination movements [31].

**Fig 3:**
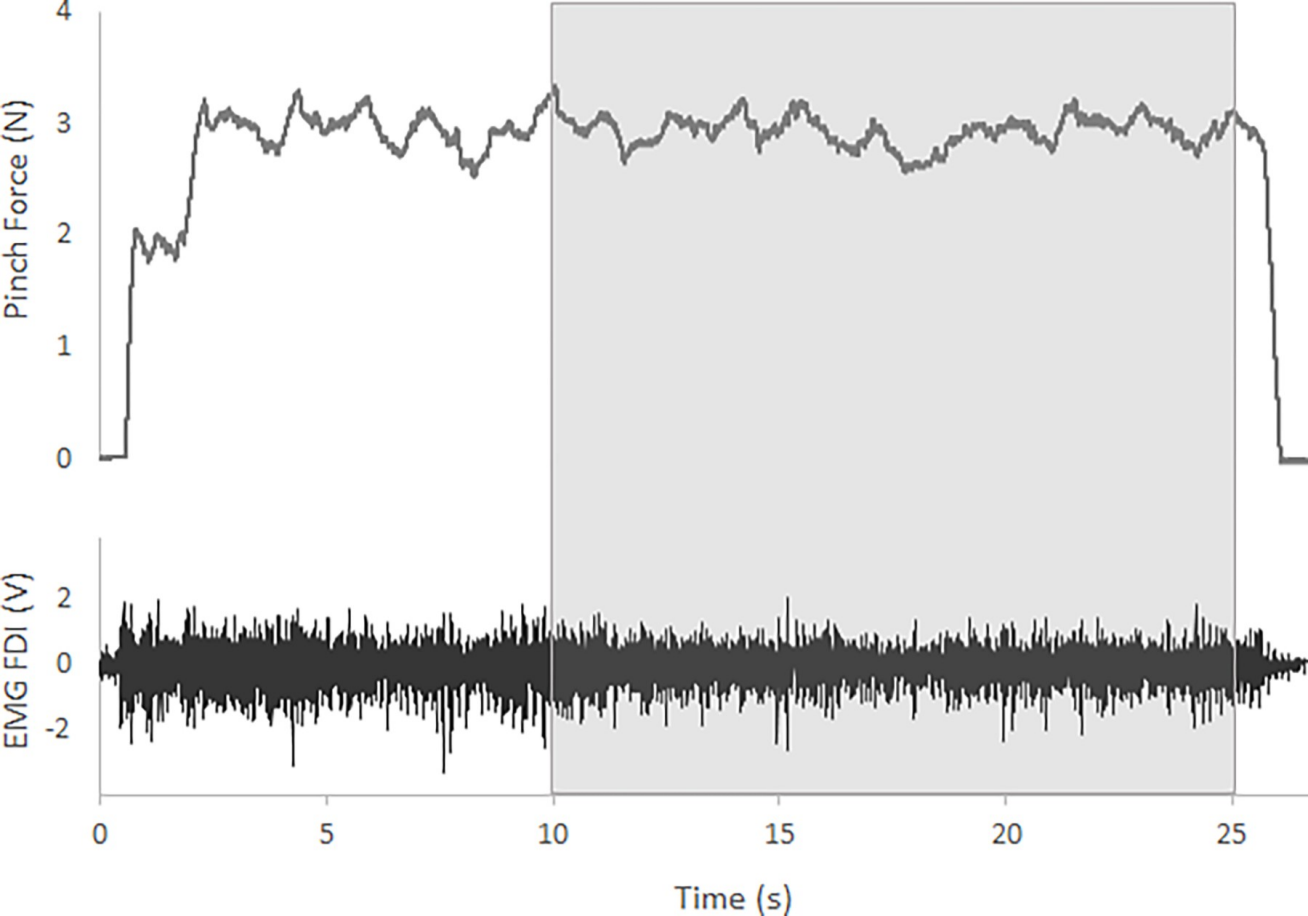
Pinch force recording sample. Force output (upper) and first dorsal interosseous EMG (lower) raw data from a participant during the pinch task at 3 N target value. The area under the grey shade is the section of data used for the analysis. As can be seen, during the first seconds there is a phase of adjustment before the volunteer was able to set the force output at the desired target. For some volunteers this adjustment phase last longer, consequently we decided to analyse exclusively the last 15 seconds of each isometric contraction.

Pinch force was analysed in term of both isometric tremor and force steadiness of the last 15 seconds of each contraction (Fig 3). Isometric tremor was measured as standard deviation (STD) of the band pass (3–30 Hz) force signal, and by looking at the position and amplitude of the dominant peak in the related power spectra. In this way it was possible to isolate the force fluctuation related exclusively to tremor. Force steadiness was quantified in terms of STD and coefficient of variation (CoV) of the raw force signal [40] and by looking at the position and amplitude of the dominant power spectra peak within a low frequency range (0–3 Hz) [41].

The EMG signals was pre-amplified (500) band-pass filtered (3–900 Hz) and A–D converted at a sampling rate of 2048 Hz. The root mean square (RMS) value of the EMG data during the last 15 s of the contraction was calculated.

Hand dexterity was assessed as contact time between the wand and the circuit during the kinetic tremor task (Fig 2A); for this evaluation the task execution time was not taken into consideration since the volunteers were only instructed not to touch the wire by choosing a speed that would have allowed him/her to avoid/minimise any contact. Finger dexterity as score in the standard 30 second, single hand, Purdue Pegboard test and as score in the 30 seconds Purdue Pegboard test performed with the tweezers.

### Statistical analysis

All measurements of tremor and steadiness were repeated twice before and twice after the protocols, and finally averaged for each individual. In preparing the data for statistical analysis of these datasets (tremor and steadiness), outliers were excluded using a median absolute deviation of two. As a robustness check, we repeated the analysis based on a less conservative median absolute deviation of three. However, the relevant significance levels and consequently their conclusions did not change. As most of these measurements were not normally distributed, that is, the standard Shapiro-Wilks test rejected the null hypothesis of normality, we applied the wild bootstrap proposed by Wu [42] for two-way ANOVA hypothesis testing (here, we use the implementation in the R-package `lmboot’). Note that this procedure also allows accounting for the problem of potential sphericity.

The results of the POMS were normally distributed, therefore the analysis was conducted with the classical two-way ANOVA (with time, group and interaction effect). For these series of data, no outliers were excluded.

Type 2 t-test was used for comparing the average values of the measurements collected during the 100-minute intervention or control protocol, additionally we made use of the longitudinal structure of the data and estimated a linear mixed-effects model [43]. Thereby, we regressed the collected values on the group (intervention or control), time (10 or 11 points in time), and their interaction, including individual-specific intercepts which deviate from a fixed common intercept. Note that the standard errors have been estimated allowing for a group-specific constant variance as a Breusch-Pagan test for heteroscedasticity suggested differences in variation across groups.

We reported the effect size for all the significant results as Hedges’ *g* when comparing only two groups (e.g. between average perceived effort during the mental task, intervention vs control group), and as partial eta-squared η^2^ for more than two sets of observations (e.g. when an interaction effect was observed) (for guidelines see [44]).

The statistical analysis was performed using R [45].

## Results

All results are presented as average ± standard deviation.

The average number of mistakes during the cognitive task was 2.8±1.7 every 100 answers. The average number of answers provided during the entire mental fatigue task was 1470±202 with an average reaction time of 1126±123 ms.

**Fig 4:**
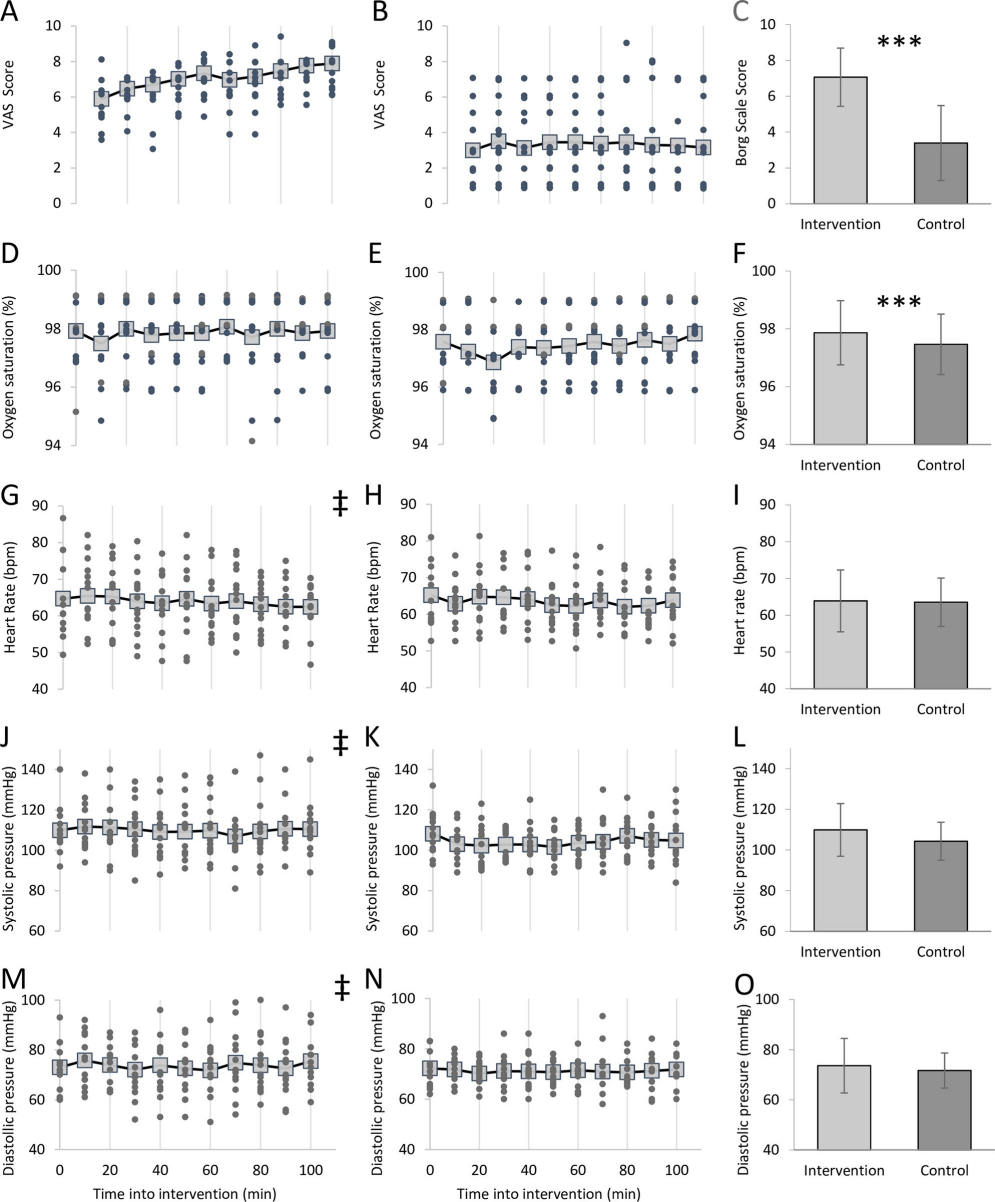
Measurements during the intervention. Individual (filled dots) and group average (transparent squares) values monitored in the intervention group (left column) and control group (middle column) for **A-C**: VAS; **D-F**: Oxygen saturation; **G-I**: Heart rate; **J-L**: Systolic pressure; **M**-**O**: Diastolic pressure. Right column group average ±SD in the intervention (light grey) and control group (dark grey). ‡p < .001 based on Breusch-Pagan test for heteroscedasticity, between **J** and **K** and between **M** and **N**. *** p < .001 based on a type 2 t-test. Hedges‘ *g* effect size: *g* = 1.97 for visual analogue scale, and *g* = 0.37 for oxygen saturation.

The results of the POMS questionnaire are presented in Table 1. A group-time interaction effect was observed for feeling of fatigue, anger, vigour and happiness (details in Table 1). The results of the mixed-effects model on the VAS assessment revealed statistical differences between the intervention and the control group starting from the third measurement (30 minutes into the intervention) until the last measurement (p < .02 for all comparisons). The estimated group-time interaction coefficient increased from 0.95 at third measurement to 2.56 at the last measurement (Fig 4A and 4B).

**Table 1:** Group average results for the POMS questionnaire before and after mental fatigue.

	Intervention			Control			Group–Time interaction	Effect size
	Pre	Post	% Change	Pre	Post	% Change		
Tension	2.2±2.7	1.1±1.8	-50.0	1.4±2.7	1.1±1.7	-21.4	F = 1.507 p = .230	
Depression	1.9±3.0	0.9±1.6	-52.6	0.7±1.3	0.7±2.1	0.0	F = 1.570 p = .221	
Anger *	1.2±1.5	3.2±2.4	+180.0	1.4±1.9	1.4±1.8	0.0	F = 9.897 *p = .004	.276
Fatigue *	4.6±3.3	9.2±3.7	+100.0	3.7±2.0	4.9±3.1	+32.4	F = 6.389 *p = .018	.191
Vigour *	11.0±3.5	6.1±3.4	-44.5	9.1±3.4	7.4±3.8	-18.7	F = 7.755 *p = .010	.223
Confusion	2.2±2.9	2.4±2.4	+9.1	2.0±2.4	1.5±1.8	-25.0	F = 0.493 p = .489	
Happiness *	8.9±2.5	5.8±3.0	-34.8	9.7±2.6	9.1±2.6	-6.2	F = 15.225 *p = .001	.361
Calmness	10.6±2.9	8.4±2.2	-20.8	10.3±2.5	10.5±3.0	+1.9	F = 3.547 p = .070	

* significant group-time interaction effect

Effect size reported as partial eta-squared η^2^ (small effect: η^2^ = 0.01, medium effect: η^2^ = 0.06, and large effect: η^2^ = 0.14) [46].

Fine manual dexterity, assessed as contact time between the wand and the circuit during the kinetic tremor task (Fig 2A) was not affected by the procedure. Average contact time was 1.46±1.23 s before and 1.07±0.92 s after (intervention group) and 1.31±1.25 s before and 0.61±0.61 s after (control group). The time for completing the circuit (about which neither restrictions nor directions were given) was also not influenced by mental fatigue and was 58.7±26.5 s before and 54.2±19.1 s after (intervention group) and 44.3±20.6 s before and 42.4±17.8 s after (control group). No correlation was observed between the time employed to complete the buzzwire and the contact time for both intervention (R^2^ = 0.12 pre, R^2^ = 0.06 post) and control (R^2^ = 0.03 pre, R^2^ = 0.07 post) groups. For finger dexterity, quantified by the score of the Purdue test performed either normally or with the tweezers, no effects for time, group or interaction were observed. Group average number of pins successfully inserted in the holes were: 17±1.8 pre and 17.1±1.9 post and 6.1±1.5 pre and 6.4±1.3 post for normal and tweezers performance respectively, in the intervention group, and 17.4±1.4 pre and 17.4±1.2 post and 6.4±1.2 pre and 6.5±1.2 post for normal and tweezers performance respectively, in the control group.

All the results related to the assessment of tremor during the postural and the goal-directed task, force steadiness and FDI EMG during the isometric pinch contractions, are presented in Table 2. None of the tested parameters (standard deviation, frequency, amplitude and area around the most prominent peak within 7–13 Hz, coefficient of variation and EMG root mean square) was different between the two groups at baseline and none was affected by the intervention (Table 2).

**Table 2:** Results of tremor and force steadiness.

		STD (force or acceleration)		PeaK (mv^2^)		Frequency (Hz)		Area (mv^2^)		EMG RMS (mv)		CoV (force)	
		Pre	Post	Pre	Post	Pre	Post	Pre	Post	Pre	Post	Pre	Post
INTERVENTION	Pinch force 3 N	7.1±2.4	7.5±2.5	7.3±5.2	8.2±6.4	7.9±0.6	8.1±0.7	6.6±4.4	6.7±5.3	0.17±0.1	0.18±0.1	0.04±0.02	0.03±0.01
	Pinch force 5 N	10.6±4.6	9.9±4.0	15.3±11.4	11.1±9.4	7.8±0.7	7.8±0.7	14.4±10.1	14.3±12.3	0.18±0.1	0.20±0.1	0.03±0.02	0.03±0.01
	Hand postural tremor	1.8±0.6	2.0±0.9	1.7±1.3	2.1±2.2	8.8±0.7	8.8±0.6	0.2±0.2	0.2±0.2				
	Arm postural tremor	2.0±0.7	2.1±0.8	0.9±0.8	1.3±1.6	9.1±0.9	9.1±1.0	0.2±0.2	0.3±0.2				
	Kinetic tremor	6.7±3.2	6.7±2.5	28.5±37.3	25.7±24.4	9.9±0.7	10.0±0.8	1.2±0.5	1.5±0.7				
CONTROL	Pinch force 3 N	9.5±3.0	9.1±2.6	16.2±14.9	12.0±9.8	8.4±0.9	8.2±1.0	9.7±5.7	8.9±6.7	0.19±0.1	0.17±0.1	0.03±0.04	0.03±0.03
	Pinch force 5 N	7.4±2.9	6.7±2.1	9.7±11.2	7.2±7.6	8.2±0.8	8.0±0.7	6.7±5.6	5.9±3.7	0.15±0.1	0.14±0.1	0.03±0.03	0.02±0.02
	Hand postural tremor	1.7±0.7	1.5±0.4	1.6±1.8	1.1±1.2	8.7±0.8	8.7±0.8	0.2±0.5	0.2±0.2				
	Arm postural tremor	1.7±0.4	1.6±0.2	0.7±0.5	0.5±0.3	9.0±0.9	9.1±0.7	0.2±0.2	0.1±0.1				
	Kinetic tremor	8.2±3.3	8.5±3.1	39.2±43.0	37.5±30.4	10.0±0.9	9.8±0.7	1.6±1.6	2.2±2.1				

Values are reported in terms of group average result ± standard deviation, for the intervention (top) and control (bottom) groups.

STD = standard deviation; Peak = amplitude of the most prominent peak within 7–13 Hz frequency band; Frequency = frequency of the most prominent peak within 7–13 Hz; Area = area within 0.5Hz of the peak; EMG RMS = root mean square of the EMG recorded from the FDI during the isometric pinch tasks; CoV = coefficient of variation of the raw force signal during the isometric pinch tasks.

Cardiovascular results are presented in Fig 4 panels D to O. Although oxygen saturation was not different between the groups at any specific time point before and during the intervention task (Fig 4D and 4E), it was on average higher in the intervention group than in the control group (p = .0002) throughout the intervention (Fig 4F). Heart rate and blood pressure were not different at baseline or any time point during the interventions (Fig 4G–4H, 4J–4K and 4N–4M) and also no differences between group averages were detected (Fig 4I, 4L and 4O). However, when looking at the individual points in Fig 4, it appears that the values in the intervention group (Fig 4 left column) are more spread compared to those in the control group (Fig 4 middle column) for heart rate and blood pressure, throughout the intervention period. A Breusch-Pagan test for heteroscedasticity was conducted to verify this observation and confirmed a difference in data variability for these parameters (p = .0009, p = .0005 and p = .00003 for heart rate, systolic pressure and diastolic pressure respectively).

## Discussion

This study aimed to assess whether mental fatigue has an effect on physiological tremor, isometric force steadiness and manual dexterity in young healthy adults. The results show that none of these parameters were affected by mental fatigue. Therefore, it is likely that neurophysiological and psychobiological alterations that are commonly observed during mental fatigue either do not influence tremor and fine manual dexterity motor control or quickly recover after it.

The effectiveness of the intervention to induce mental fatigue was confirmed by a subjective report of a considerable increase in feelings of fatigue (+100%) together with a 44.5% decrease in vigour and a parallel increase of perceived effort during the execution of the mental task (Fig 4A) that are typical characterizations of a cognitive fatigued state [3, 47].

Other indicators of the effort in having to continue the mental protocol, can be inferred by the different levels of oxygen saturations between the intervention and control groups during the respective tasks (Fig 4F). Indeed, a normal oxygen saturation level is required to maintain cognitive performance [48] and it raises with increased requirements of cognitive control, as for example during a conflict task like the one used in our study [49]. Finally, the bigger variation of heart rate, that is a recognised index of mental effort [50], and blood pressure within the intervention group might suggest a psychophysiological reaction to the cognitive task that could have increased the levels of stress and alertness in some of the participants and decreased in others [51] without affecting the volunteers in the control group, who remained similarly neutral to their assigned task, as corroborated by a decrease in feelings of happiness and an increase in anger in the intervention, but not in the control group

### Force steadiness and EMG

In our experiment force steadiness was not affected by mental fatigue at neither of the two absolute isometric contraction intensities tested. Our result is supported by previous reports showing that isometric force steadiness tested following a mental effort was not affected in neither the lower [28] nor the upper body [27]. Differently, a declines in force steadiness has been reported during mental efforts [22, 26, 52], however, the simultaneous execution of two different tasks (cognitive and physical) generates the so call cognitive motor interference [53–55] that is a phenomenon not comparable to mental fatigue. The corresponding EMG activity recorded from the FDI during the pinch tasks was also unaffected by the intervention. Similar results were presented by others [11, 27] and additionally, mental fatigue was reported not to affect M wave [56] or motor unit firing frequency [28]. Nevertheless, several studies reported an increase in muscular activity during an isometric endurance handgrip following a cognitive effort [57–59]. However, having recorded the EMG from the FDI gives us a more controlled condition compared to testing a forearm muscle surrounded by several synergists (as in [57–59]) that might create noise in the recorded signal, as also acknowledged by the authors themselves [57]. Additionally, Brown and Bray [59] showed that the increase in EMG activity was substantially attenuated when the volunteers were given monetary incentives to continue the endurance handgrip task. This suggests that motivation in sustaining an effort influences muscular activation, with lower enthusiasm being related to stronger activation. Our task was of short duration (25 seconds) and required minimal effort (3 or 5 N), this might have prevented this low-motivation-induced increase in the EMG signal reported by others [59].

### Dexterity

Functional alterations in anterior cingulate cortex and striatum have been reported during prolonged cognitive tasks [60, 61] and during sleep deprivation [62]. Both these regions of the brain are involved in motor learning and motor control [63, 64] and, accordingly, a malfunctioning or a hypoactivity of these areas have been associated with motor learning impairment [65], diminished neural capacity to detect errors [66] and dysfunction in the motor control system [67]. Based on these findings a reduction in fine movement control could be expected to mirror a state of mental fatigue. Our results contradict this expectation, but it has to be pointed out that the functional activity in the anterior cingulate cortex and striatum was reported to be altered during and not after mental fatigue [60, 61]. This might suggest that any reorganisation and/or impairments of functional brain activities during a mental task are reversed soon after the cognitive effort is interrupted without any long-lasting effect being detectable. In support of this short-term effect suggestion, an animal study demonstrated that glucose in the brain is compartmentalized and it is temporarily withdrawn from not active areas only for the duration of a mental task for then returning to the baseline levels [68].

In disagreement with this explanation and with our results though, Valenza and colleagues (2020) reported a decrease in manual dexterity following mental fatigue. Differently to Valenza et al. (2020), who adopted an O’Connor dexterity test (that involves the insertion of 300 pins in 100 holes) and a 3 minutes version of a Hand-Tool Dexterity test, we used much shorter tests: our Purdue and the isometric pinch tests lasted 30 seconds, and the buzz wire circuit, on average, less than a minute. Such different demand/complexity of the task used for assessing dexterity is a crucial aspect that needs to be discussed. Indeed, studies on the effects of mental fatigue on gait parameters demonstrated that mental fatigue affected dual task gait (walking during the execution of arithmetic calculation) but did not affect single task gait (just walking) [15]. Similarly, an impairment in performance was observed in fatigued subjects when executing a variety of motor tasks and sport-technical skills (like football [69], table tennis [70] and cricket [71]), but not when simple isolated (e.g. isometric contractions) or all out simple whole-body (e.g. sprint running, all-out cycling, jumping) exercises were tested (for review see [11]. It is therefore necessary to acknowledge the limited ecological validity of our testing procedure, because, being it based on simple single tasks, does not replicate sport and professional scenarios requiring fine motor control which usually involve a significant and concurrent cognitive load.

### Tremor

As mentioned in the introduction, to our knowledge, a direct examination of the effects of mental fatigue on tremor was reported only by Budini et al. (2014a). The authors [6] observed a decrease in the amplitude of mechanically amplified tremor within 8–12 Hz and attributed this result to first having induced cortico-muscular coherence within this frequency band during the contractions against the spring [72] and subsequently having cancelled it by inducing mental fatigue. However, because in healthy individuals, cortico-muscular coherence within the frequency band of physiological tremor is not commonly observed [72–74] this result offers limited possibility of comparison with the current data.

Indirect evidence of the possible relationship between mental fatigue and tremor can be inferred by linking the increased levels of cortisol and catecholamine reported during continuous mental task [75, 76] with worsened tremor [77, 78] as well as exacerbated low frequency oscillations during sustained pinch-grip force [79] when assessed at high plasma concentrations of these hormones. Contrary to what suggested for the functional alterations of brain regions, where the effects of mental fatigue seem to be reversed as soon as the cognitive effort terminates, higher adrenaline values can persist for hours following the intervention [75]. However, the amount of the circulating hormone in response to mental fatigue is likely not sufficient to induce an effect on muscle tremor [77, 80].

## Conclusion

One-hundred minutes of continuative cognitive task induced mental fatigue but this did not influence force steadiness, EMG, hand dexterity and muscle tremor. It is possible that the alterations commonly observed during mental fatigue and that could have influenced the parameters we measured only last for the duration of the cognitive task and are not detectable anymore soon after the mental task is terminated. It cannot be excluded though that a different, more demanding intervention, might induce more long-lasting detectable effects or that a different testing procedure requiring a dual task effort might highlight an effect of mental fatigue on dexterity and tremor that we could identify with our testing procedures.

## Supporting information

S1 File Cardio vascular data. (XLSX)

S2 File Data for postural, kinetic and isometric tremor, and EMG. Control group.
(XLSX)

S3 File Data for postural, kinetic and isometric tremor, and EMG. Intervention group.
(XLSX)

S4 File POMS scores. (XLSX)

S5 File Buzzwire contact time. (XLS)

## References

[1] Lal SK, Craig A. Driver fatigue: electroencephalography and psychological assessment. Psychophysiology. 2002;39: 313–321. doi: 10.1017/s0048577201393095 12212650

[2] Zhao C, Zhao M, Liu J, Zheng C. Electroencephalogram and electrocardiograph assessment of mental fatigue in a driving simulator. Accid Anal Prev. 2012;45: 83–90. doi: 10.1016/j.aap.2011.11.019 22269488

[3] Sasahara I, Fujimura N, Nozawa Y, Furuhata Y, Sato H. The effect of histidine on mental fatigue and cognitive performance in subjects with high fatigue and sleep disruption scores. Physiol Behav. 2015;147: 238–244. doi: 10.1016/j.physbeh.2015.04.042 25921948

[4] Lorist MM, Klein M, Nieuwenhuis S, De Jong R, Mulder G, Meijman TF. Mental fatigue and task control: planning and preparation. Psychophysiology. 2000;37: 614–625. 11037038

[5] Langner R, Steinborn MB, Chatterjee A, Sturm W, Willmes K. Mental fatigue and temporal preparation in simple reaction-time performance. Acta Psychol (Amst). 2010;133: 64–72. doi: 10.1016/j.actpsy.2009.10.001 19878913

[6] Budini F, Lowery M, Durbaba R, De Vito G. Effect of mental fatigue on induced tremor in human knee extensors. J Electromyogr Kinesiol. 2014;24: 412–418. doi: 10.1016/j.jelekin.2014.02.003 24613661

[7] Boksem MA, Meijman TF, Lorist MM. Effects of mental fatigue on attention: an ERP study. Cogn Brain Res. 2005;25: 107–116. doi: 10.1016/j.cogbrainres.2005.04.011 15913965

[8] Hopstaken JF, van der Linden D, Bakker AB, Kompier MA, Leung YK. Shifts in attention during mental fatigue: Evidence from subjective, behavioral, physiological, and eye-tracking data. J Exp Psychol Hum Percept Perform. 2016;42: 878. doi: 10.1037/xhp0000189 26752733

[9] Lim J, Dinges D. Sleep deprivation and vigilant attention. Ann N Y Acad Sci. 2008;1129: 305. doi: 10.1196/annals.1417.002 18591490

[10] Van Cutsem J, Marcora S, De Pauw K, Bailey S, Meeusen R, Roelands B. The effects of mental fatigue on physical performance: a systematic review. Sports Med. 2017;47: 1569–1588. doi: 10.1007/s40279-016-0672-0 28044281

[11] Pageaux B, Lepers R. The effects of mental fatigue on sport-related performance. Progress in brain research. Elsevier; 2018. pp. 291–315. doi: 10.1016/bs.pbr.2018.10.004 30390836

[12] Brown DM, Graham JD, Innes KI, Harris S, Flemington A, Bray SR. Effects of prior cognitive exertion on physical performance: A systematic review and meta-analysis. Sports Med. 2020;50: 497–529. doi: 10.1007/s40279-019-01204-8 31873926

[13] Borghini G, Vecchiato G, Toppi J, Astolfi L, Maglione A, Isabella R, et al. Assessment of mental fatigue during car driving by using high resolution EEG activity and neurophysiologic indices. 2012 Annual International Conference of the IEEE Engineering in Medicine and Biology Society; 2012. pp. 6442–6445. doi: 10.1109/EMBC.2012.6347469 23367404

[14] Lew FL, Qu X. Effects of mental fatigue on biomechanics of slips. Ergonomics. 2014;57: 1927–1932. doi: 10.1080/00140139.2014.937771 25017252

[15] Behrens M, Mau-Moeller A, Lischke A, Katlun F, Gube M, Zschorlich V, et al. Mental fatigue increases gait variability during dual-task walking in old adults. J Gerontol Ser A. 2018;73: 792–797. doi: 10.1093/gerona/glx210 29077783

[16] Hachard B, Noé F, Ceyte H, Trajin B, Paillard T. Balance control is impaired by mental fatigue due to the fulfilment of a continuous cognitive task or by the watching of a documentary. Exp Brain Res. 2020; 1–8. doi: 10.1007/s00221-020-05758-2 32146502

[17] Qu X, Xie Y, Hu X, Zhang H. Effects of fatigue on balance recovery from unexpected trips. Hum Factors. 2020;62: 919–927. doi: 10.1177/0018720819858794 31385721

[18] Tassignon B, Verschueren J, De Pauw K, Roelands B, Van Cutsem J, Verhagen E, et al. Mental fatigue impairs clinician‐friendly balance test performance and brain activity. Transl Sports Med. 2020;3: 616–625.

[19] Varas-Diaz G, Kannan L, Bhatt T. Effect of mental fatigue on postural sway in healthy older adults and stroke populations. Brain Sci. 2020;10: 388. doi: 10.3390/brainsci10060388 32575383 PMC7349503

[20] Engelmann C, Schneider M, Kirschbaum C, Grote G, Dingemann J, Schoof S, et al. Effects of intraoperative breaks on mental and somatic operator fatigue: a randomized clinical trial. Surg Endosc. 2011;25: 1245–1250. doi: 10.1007/s00464-010-1350-1 20835716

[21] Sugden C, Athanasiou T, Darzi A. What are the effects of sleep deprivation and fatigue in surgical practice? Seminars in thoracic and cardiovascular surgery (Vol. 24, No. 3, pp. 166–175). WB Saunders.; 2012. pp. 166–175. doi: 10.1053/j.semtcvs.2012.06.005 23200071

[22] Vanden Noven ML, Pereira HM, Yoon T, Stevens AA, Nielson KA, Hunter SK. Motor variability during sustained contractions increases with cognitive demand in older adults. Front Aging Neurosci. 2014;6: 97. doi: 10.3389/fnagi.2014.00097 24904410 PMC4033244

[23] Pereira HM, Spears VC, Schlinder-Delap B, Yoon T, Nielson KA, Hunter SK. Age and sex differences in steadiness of elbow flexor muscles with imposed cognitive demand. Eur J Appl Physiol. 2015;115: 1367–1379. doi: 10.1007/s00421-015-3113-0 25633070 PMC4431934

[24] Pereira HM, Spears VC, Schlinder-Delap B, Yoon T, Harkins A, Nielson KA, et al. Sex differences in arm muscle fatigability with cognitive demand in older adults. Clin Orthop Relat Res. 2015;473: 2568–2577. doi: 10.1007/s11999-015-4205-1 25712862 PMC4488210

[25] Pereira HM, Schlinder-Delap B, Nielson KA, Hunter SK. Force steadiness during a cognitively challenging motor task is predicted by executive function in older adults. Front Physiol. 2018;9: 1316. doi: 10.3389/fphys.2018.01316 30333758 PMC6176355

[26] Pereira HM, Schlinder-DeLap B, Keenan KG, Negro F, Farina D, Hyngstrom AS, et al. Oscillations in neural drive and age-related reductions in force steadiness with a cognitive challenge. J Appl Physiol. 2019;126: 1056–1065. doi: 10.1152/japplphysiol.00821.2018 30817244 PMC6485692

[27] Shortz AE, Mehta RK. Cognitive challenges, aging, and neuromuscular fatigue. Physiol Behav. 2017;170: 19–26. doi: 10.1016/j.physbeh.2016.11.034 27894796

[28] Kowalski KL, Anita D C. Force Control and Motor Unit Firing Behavior Following Mental Fatigue in Young Female and Male Adults. Front Integr Neurosci. 2020;14: 15. doi: 10.3389/fnint.2020.00015 32296312 PMC7137823

[29] Luck O, Reitemeier B, Scheuch K. Testing of fine motor skills in dental students. Eur J Dent Educ. 2000;4: 10–14. doi: 10.1034/j.1600-0579.2000.040103.x 11168460

[30] Aaron DH, Jansen CWS. Development of the Functional Dexterity Test (FDT): construction, validity, reliability, and normative data. J Hand Ther. 2003;16: 12–21. doi: 10.1016/s0894-1130(03)80019-4 12611441

[31] Budini F, Lowery MM, Hutchinson M, Bradley D, Conroy L, De Vito G. Dexterity training improves manual precision in patients affected by essential tremor. Arch Phys Med Rehabil. 2014;95: 705–710. doi: 10.1016/j.apmr.2013.11.002 24275064

[32] Rozand V, Lebon F, Papaxanthis C, Lepers R. Effect of mental fatigue on speed–accuracy trade-off. Neuroscience. 2015;297: 219–230. doi: 10.1016/j.neuroscience.2015.03.066 25849613

[33] Duncan MJ, Fowler N, George O, Joyce S, Hankey J. Mental fatigue negatively influences manual dexterity and anticipation timing but not repeated high-intensity exercise performance in trained adults. Res Sports Med. 2015;23: 1–13. doi: 10.1080/15438627.2014.975811 25630242

[34] Valenza A, Charlier H, Bianco A, Filingeri D. Independent and interactive effects of thermal stress and mental fatigue on manual dexterity. Am J Physiol-Regul Integr Comp Physiol. 2020;319: R703–R711. doi: 10.1152/ajpregu.00226.2020 33074012

[35] Culver DH, Horan TC, Gaynes RP, Martone WJ, Jarvis WR, Emori TG, et al. Surgical wound infection rates by wound class, operative procedure, and patient risk index. Am J Med. 1991;91: S152–S157. 10.1016/0002-9343(91)90361-z 1656747

[36] Frese EM, Fick A, Sadowsky HS. Blood pressure measurement guidelines for physical therapists. Cardiopulm Phys Ther J. 2011;22: 5. 21637392 PMC3104931

[37] Curran SL, Andrykowski MA, Studts JL. Short form of the profile of mood states (POMS-SF): psychometric information. Psychol Assess. 1995;7: 80.

[38] Quartiroli A, Terry PC, Fogarty GJ. Development and initial validation of the Italian Mood Scale (ITAMS) for use in sport and exercise contexts. Front Psychol. 2017;8: 1483. doi: 10.3389/fpsyg.2017.01483 28936185 PMC5594224

[39] Budini F, Laudani L, Bernardini S, Macaluso A. Local vibration inhibits H-reflex but does not compromise manual dexterity and does not increase tremor. Hum Mov Sci. 2017;55: 221–228. doi: 10.1016/j.humov.2017.08.018 28843638

[40] Bazzucchi I, Felici F, Macaluso A, De Vito G. Differences between young and older women in maximal force, force fluctuations, and surface EMG during isometric knee extension and elbow flexion. Muscle Nerve. 2004;30: 626–635. doi: 10.1002/mus.20151 15389720

[41] Lodha N, Christou EA. Low-frequency oscillations and control of the motor output. Front Physiol. 2017;8: 78. doi: 10.3389/fphys.2017.00078 28261107 PMC5306248

[42] Wu C-FJ. Jackknife, bootstrap and other resampling methods in regression analysis. Ann Stat. 1986;14: 1261–1295.

[43] Gałecki A, Burzykowski T. Linear mixed-effects model. Linear Mixed-Effects Models Using R. Springer; 2013. pp. 245–273.

[44] Lakens D. Calculating and reporting effect sizes to facilitate cumulative science: a practical primer for t-tests and ANOVAs. Front Psychol. 2013;4: 863. doi: 10.3389/fpsyg.2013.00863 24324449 PMC3840331

[45] Team RC. R: A language and environment for statistical computing. 2013.

[46] Cohen J. Statistical power analysis for the behavioral sciences New York. NY Acad. 1988; 54.

[47] Vrijkotte S, Meeusen R, Vandervaeren C, Buyse L, Van Cutsem J, Pattyn N, et al. Mental fatigue and physical and cognitive performance during a 2-bout exercise test. Int J Sports Physiol Perform. 2018;13: 510–516. doi: 10.1123/ijspp.2016-0797 28952829

[48] Williams TB, Corbett J, McMorris T, Young JS, Dicks M, Ando S, et al. Cognitive performance is associated with cerebral oxygenation and peripheral oxygen saturation, but not plasma catecholamines, during graded normobaric hypoxia. Exp Physiol. 2019;104: 1384–1397. doi: 10.1113/EP087647 31192502

[49] León-Carrion J, Damas-López J, Martín-Rodríguez JF, Domínguez-Roldán JM, Murillo-Cabezas F, y Martin JMB, et al. The hemodynamics of cognitive control: the level of concentration of oxygenated hemoglobin in the superior prefrontal cortex varies as a function of performance in a modified Stroop task. Behav Brain Res. 2008;193: 248–256. doi: 10.1016/j.bbr.2008.06.013 18606191

[50] Mukherjee S, Yadav R, Yung I, Zajdel DP, Oken BS. Sensitivity to mental effort and test–retest reliability of heart rate variability measures in healthy seniors. Clin Neurophysiol. 2011;122: 2059–2066. doi: 10.1016/j.clinph.2011.02.032 21459665 PMC3132243

[51] Matthews KA, Woodall KL, Allen MT. Cardiovascular reactivity to stress predicts future blood pressure status. Hypertension. 1993;22: 479–485. doi: 10.1161/01.hyp.22.4.479 8406652

[52] Lorist MM, Kernell D, Meijman TF, Zijdewind I. Motor fatigue and cognitive task performance in humans. J Physiol. 2002;545: 313–319. doi: 10.1113/jphysiol.2002.027938 12433971 PMC2290666

[53] Al-Yahya E, Dawes H, Smith L, Dennis A, Howells K, Cockburn J. Cognitive motor interference while walking: a systematic review and meta-analysis. Neurosci Biobehav Rev. 2011;35: 715–728. doi: 10.1016/j.neubiorev.2010.08.008 20833198

[54] Leone C, Feys P, Moumdjian L, D’Amico E, Zappia M, Patti F. Cognitive-motor dual-task interference: a systematic review of neural correlates. Neurosci Biobehav Rev. 2017;75: 348–360. doi: 10.1016/j.neubiorev.2017.01.010 28104413

[55] Bank PJ, Marinus J, van Tol RM, Groeneveld IF, Goossens PH, de Groot JH, et al. Cognitive‐motor interference during goal‐directed upper‐limb movements. Eur J Neurosci. 2018;48: 3146–3158. doi: 10.1111/ejn.14168 30251278 PMC6282826

[56] Morris AJ, Christie AD. The effect of mental fatigue on neuromuscular function is similar in young and older women. Brain Sci. 2020;10: 191. doi: 10.3390/brainsci10040191 32218178 PMC7226096

[57] Bray SR, Martin Ginis KA, Hicks AL, Woodgate J. Effects of self‐regulatory strength depletion on muscular performance and EMG activation. Psychophysiology. 2008;45: 337–343. doi: 10.1111/j.1469-8986.2007.00625.x 17995906

[58] Graham JD, Sonne MW, Bray SR. It wears me out just imagining it! Mental imagery leads to muscle fatigue and diminished performance of isometric exercise. Biol Psychol. 2014;103: 1–6. doi: 10.1016/j.biopsycho.2014.07.018 25093627

[59] Brown DM, Bray SR. Effects of mental fatigue on physical endurance performance and muscle activation are attenuated by monetary incentives. J Sport Exerc Psychol. 2017;39: 385–396. doi: 10.1123/jsep.2017-0187 29424609

[60] Lorist MM, Boksem MA, Ridderinkhof KR. Impaired cognitive control and reduced cingulate activity during mental fatigue. Cogn Brain Res. 2005;24: 199–205. doi: 10.1016/j.cogbrainres.2005.01.018 15993758

[61] Lim J, Wu W, Wang J, Detre JA, Dinges DF, Rao H. Imaging brain fatigue from sustained mental workload: an ASL perfusion study of the time-on-task effect. Neuroimage. 2010;49: 3426–3435. doi: 10.1016/j.neuroimage.2009.11.020 19925871 PMC2830749

[62] Fang Z, Spaeth AM, Ma N, Zhu S, Hu S, Goel N, et al. Altered salience network connectivity predicts macronutrient intake after sleep deprivation. Sci Rep. 2015;5: 1–8. doi: 10.1038/srep08215 25645575 PMC4314629

[63] Jueptner M, Stephan KM, Frith CD, Brooks DJ, Frackowiak RS, Passingham RE. Anatomy of motor learning. I. Frontal cortex and attention to action. J Neurophysiol. 1997;77: 1313–1324. doi: 10.1152/jn.1997.77.3.1313 9084599

[64] Wächter T, Röhrich S, Frank A, Molina-Luna K, Pekanovic A, Hertler B, et al. Motor skill learning depends on protein synthesis in the dorsal striatum after training. Exp Brain Res. 2010;200: 319–323. doi: 10.1007/s00221-009-2027-7 19823812

[65] Lemay-Clermont J, Robitaille C, Auberson YP, Bureau G, Cyr M. Blockade of NMDA receptors 2A subunit in the dorsal striatum impairs the learning of a complex motor skill. Behav Neurosci. 2011;125: 714. doi: 10.1037/a0025213 21859173

[66] Hester R, Nestor L, Garavan H. Impaired error awareness and anterior cingulate cortex hypoactivity in chronic cannabis users. Neuropsychopharmacology. 2009;34: 2450–2458. doi: 10.1038/npp.2009.67 19553917 PMC2743772

[67] Ridderinkhof KR, Ullsperger M, Crone EA, Nieuwenhuis S. The role of the medial frontal cortex in cognitive control. Science. 2004;306: 443–447. doi: 10.1126/science.1100301 15486290

[68] McNay EC, McCarty RC, Gold PE. Fluctuations in brain glucose concentration during behavioral testing: dissociations between brain areas and between brain and blood. Neurobiol Learn Mem. 2001;75: 325–337. doi: 10.1006/nlme.2000.3976 11300738

[69] Smith MR, Zeuwts L, Lenoir M, Hens N, De Jong LM, Coutts AJ. Mental fatigue impairs soccer-specific decision-making skill. J Sports Sci. 2016;34: 1297–1304. doi: 10.1080/02640414.2016.1156241 26949830

[70] Le Mansec Y, Pageaux B, Nordez A, Dorel S, Jubeau M. Mental fatigue alters the speed and the accuracy of the ball in table tennis. J Sports Sci. 2018;36: 2751–2759. doi: 10.1080/02640414.2017.1418647 29260619

[71] Veness D, Patterson SD, Jeffries O, Waldron M. The effects of mental fatigue on cricket-relevant performance among elite players. J Sports Sci. 2017;35: 2461–2467. doi: 10.1080/02640414.2016.1273540 28092208

[72] Budini F, McManus LM, Berchicci M, Menotti F, Macaluso A, Di Russo F, et al. Alpha band cortico-muscular coherence occurs in healthy individuals during mechanically-induced tremor. PloS One. 2014;9: e115012. doi: 10.1371/journal.pone.0115012 25514444 PMC4267728

[73] Hari R, Salenius S. Rhythmical corticomotor communication. Neuroreport. 1999;10: R1–10. 10203308

[74] Kilner JM, Baker SN, Salenius S, Hari R, Lemon RN. Human cortical muscle coherence is directly related to specific motor parameters. J Neurosci. 2000;20: 8838–8845. doi: 10.1523/JNEUROSCI.20-23-08838.2000 11102492 PMC6773054

[75] Frankenhaeuser M, Johansson G. Task demand as reflected in catecholamine excretion and heart rate. J Human Stress. 1976;2: 15–23. doi: 10.1080/0097840X.1976.9937485 1018111

[76] Bohnen N, Houx P, Nicolson N, Jolles J. Cortisol reactivity and cognitive performance in a continuous mental task paradigm. Biol Psychol. 1990;31: 107–116. doi: 10.1016/0301-0511(90)90011-k 2103746

[77] Marshall J, Schnieden H. Effect of adrenaline, noradrenaline, atropine, and nicotine on some types of human tremor. J Neurol Neurosurg Psychiatry. 1966;29: 214. doi: 10.1136/jnnp.29.3.214 5937635 PMC496021

[78] Marsden C, Meadows J. The effect of adrenaline on the contraction of human muscle. J Physiol. 1970;207: 429–448. doi: 10.1113/jphysiol.1970.sp009071 5499029 PMC1348716

[79] Christou EA, Jakobi JM, Critchlow A, Fleshner M, Enoka RM. The 1-to 2-Hz oscillations in muscle force are exacerbated by stress, especially in older adults. J Appl Physiol. 2004;97: 225–235. doi: 10.1152/japplphysiol.00066.2004 15220319

[80] Marsden C, Meadows J, Lange G, Watson R. Effect of deafferentation on human physiological tremor. The Lancet. 1967;290: 700–702. doi: 10.1016/s0140-6736(67)90977-4 4167100

